# Magnetic Properties of Silicon Steel after Plastic Deformation

**DOI:** 10.3390/ma13194361

**Published:** 2020-09-30

**Authors:** Andries Daem, Peter Sergeant, Luc Dupré, Somsubhro Chaudhuri, Vitaliy Bliznuk, Leo Kestens

**Affiliations:** 1 Department of Electromechanical, Systems and Metal Engineering, Ghent University, B-9000 Ghent, Belgium; Peter.Sergeant@UGent.be (P.S.); Luc.Dupre@UGent.be (L.D.); Somsubhro.Chaudhuri@UGent.be (S.C.); Vitaliy.Bliznuk@UGent.be (V.B.); Leo.Kestens@UGent.be (L.K.); 2 Flanders Make@UGent, Core Lab EEDT, 9000 Ghent, Belgium

**Keywords:** soft magnetic materials, magnetic material properties, core energy loss, plastic deformation, core loss separation

## Abstract

The energy efficiency of electric machines can be improved by optimizing their manufacturing process. During the manufacturing of ferromagnetic cores, silicon steel sheets are cut and stacked. This process introduces large stresses near cutting edges. The steel near cutting edges is in a plastically deformed stress state without external mechanical load. The magnetic properties of the steel in this stress state are investigated using a custom magnetomechanical measurement setup, stress strain measurements, electrical resistance measurements, and transmission electron microscopic (TEM) measurements. Analysis of the core energy losses is done by means of the loss separation technique. The silicon steel used in this paper is non-grain oriented (NGO) steel grade M270-35A. Three differently cut sets of M270-35A are investigated, which differ in the direction they are cut with respect to the rolling direction. The effect of sample deformation was measured—both before and after mechanical load release—on the magnetization curve and total core energy losses. It is known that the magnetic properties dramatically degrade with increasing sample deformation under mechanical load. In this paper, it was found that when the mechanical load is released, the magnetic properties degrade even further. Loss separation analysis has shown that the hysteresis loss is the main contributor to the additional core losses due to sample deformation. Releasing the mechanical load increased the hysteresis loss up to 270% at 10.4% pre-release strain. At this level of strain, the relative magnetic permeability decreased up to 45% after mechanical load release. Manufacturing processes that introduce plastic deformation are detrimental to the local magnetic material properties.

## 1. Introduction

**Figure 1:**
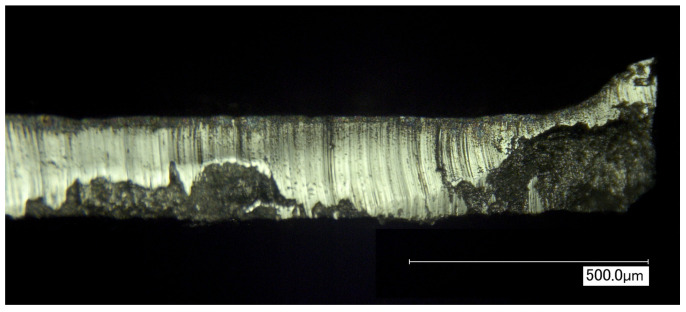
Microscopic photograph of the side view of a punched electrical steel lamination with edge burr on the right hand side. This photo was taken using a Keyence digital microscope with 250 times magnification.

Due to increasingly stringent CO_2_ emission targets, there is a global interest for improving the efficiency of electrical machines such as transformers and electric motors. When the goal is to increase efficiency, it is necessary to maintain a broad view of the development of these machines. In this paper, the focus is on a specific part of the manufacturing process of the ferromagnetic core. During the manufacturing of electrical machines, silicon steel sheets are processed using separation and joining techniques to shape and assemble the laminated ferromagnetic core. Commonly, the core laminations are cut out of the mother coil using punching, laser cutting or water jet cutting. After the separation stage, the laminations are joined to form stacks using welding, interlocking or glue bonding. The manufacturing techniques used for the separation and joining of the laminations are known to have a degrading effect on the soft magnetic qualities of the silicon steel [1,2,3]. In [4], the effect of different cutting techniques on the magnetic properties of non-oriented electrical steels was measured. It was observed that the mechanical or thermal stresses induced by the cutting techniques increase the total core energy loss and decrease the magnetic permeability. The degradation of magnetic properties can be attributed to the introduction of stress. The residual stress present in the material after cutting was measured locally near the cutting edge in [5], and near welding seams in [6]. In both cases, it was observed that the residual stress changed drastically near the locally impacted area. In mass production, punching is the most frequently applied technique for shaping the lamination geometry. Inherent to the punching process, the material on the cut edge of the steel has undergone an external mechanical load greater than the breaking point of the material. After the punching operation, the area on the edge of the sheet is in a plastically deformed stress state without external mechanical load. In Figure 1, a micrograph of the cutting edge of a punched lamination is shown, where the edge burr—which is a result of the punching process—is clearly visible. Typically, machine designers use magnetic material models obtained from data sheets or from standardized Epstein frame tests. However, when designing high efficiency electric machines, the effects of manufacturing should not be neglected. Therefore, it is in the interest of machine designers to have accurate magnetic material models at their disposal. These models should account for the degrading effects of manufacturing so that the machine model corresponds more accurately to the actual machine after production.

It is well known in literature that the magnetic properties of silicon steel can be impacted significantly by the induced stress due to the cutting process. Many researchers have proposed techniques for modelling cutting effects in electrical machines. A literature review of cutting models was given by Bali and Muetze [7]. The influence of residual stress is considered only in a small subset of the proposed cutting models, and the models that do incorporate the effect of stress in the cutting model do not consider the effect of different frequencies of the applied magnetic field. Unrelated to the cutting models, material models have been proposed that correlate the applied mechanical stress to the magnetic properties of silicon steels for uni-directional magnetization [8,9,10,11,12,13,14,15] and multi-directional magnetization [16,17,18,19]. These models are based on measurements of the effect of externally applied stress within the elastic or plastic region while external mechanical load was present. In the elastic region, the magnetic properties are restored when the mechanical load is released. In the plastic region, the microstructure is irreversibly altered. In order to facilitate useful modelling of these multi-directional effects, equivalent stress models were proposed [20,21]. The effect of compressive stress in the thickness direction was investigated similarily in [22,23,24]. Few reports where found by the authors on the effects of plastic deformation after the mechanical load was released. In [25], Sablik et al. proposed a model based on a comparison between several *BH* loops with the same peak flux density *B_p_* and a linear relation was found between residual strain and the coercive field *H_c_*. Only few reports are available containing microstructural measurements of plastically deformed electrical steel [26,27]. Makar et al. [26] performed magnetic measurements on several steel samples with different carbon content after the mechanical load has been released and studied parameters such as magnetostriction and differential permeability. Makar concluded that the changes in magnetic properties are primarily caused by the creation of pinning sites during deformation. Iordache et al. [28] reported similar measurements and assigned the degrading effects of residual strain to the appearance of long range internal stresses during strain hardening. Xiong et al. [29] investigated multiple parameters related to texture in mechanically cut samples of electrical steel and found that the dislocation density and microhardness increases exponentially near the cut edge.

In this paper, the influence of mechanical load release on the magnetic properties of silicon steel is studied to demonstrate the importance of having correct magnetomechanical data for developing manufacturing effect models. Non-linear *BH* characteristics and core loss measurements are reported, both before and after the mechanical load within the plastic region was released. This was done on samples that were cut in 3 different directions with respect to the rolling direction (RD). Also, the electrical resistivity was measured for samples with different degrees of strain after load release. The core losses were measured on a wide range of *B_p_* levels and frequencies in order to properly execute the method of core loss separation. This way, the effect of loaded and unloaded plastic deformation on each core loss component can be investigated. The results of core loss separation, together with the *BH* characteristics, can be used to increase the accuracy of cutting effect models because they are more closely related to the real state of the material after plastic deformation, which is the case near cutting edges and other locally stressed areas. Lastly, the dislocation density in the unloaded deformed samples is determined using micrographs obtained from a TEM. These microstructural properties can be correlated to the observed changes in magnetic properties.

## 2. Materials and Methods

### 2.1. M270-35A Electrical Steel Grade

The measurements and analysis reported in this paper are obtained using samples of non grain oriented silicon steel (NGO) M270-35A electrical steel. This grade has an electrical resistivity of 52 μΩ cm and sheet thickness of 0.35 mm. The samples were cut in rectangular strips of 30 mm by 300 mm using guillotine cutting. All the samples originated from the same mother coil, so that any variability in measurements was related to the applied load. Although the steel grade considered in this experiment is non grain oriented, the magnetic properties are not isotropic. There can be a significant difference between the magnetic properties measured in rolling direction (RD) and the orthogonal transverse direction (TD). The causes of this phenomenon are described in more detail in [30]. Therefore, three sets of samples were cut out in order to investigate the relation between the direction of strain, magnetic flux and anisotropy of NGO steel. One set of samples was cut in RD, one set was cut in TD, and another set was cut in diagonal direction (DD). The latter samples are cut along a 45° angle with respect to RD.

### 2.2. Magnetomechanical Setup Using Stress Bench

**Figure 2:**
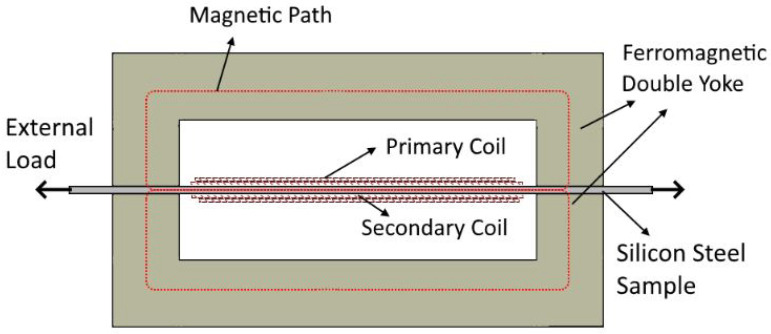
Illustrated cross section view of the double yoke setup used for performing magnetic measurements.

**Figure 3:**
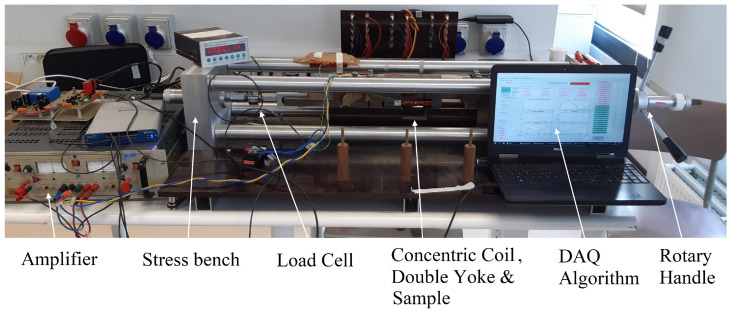
Experimental magnetomechanical setup used for magnetic measurements under external load.

Similar to the setup in [4], the magnetomechanical setup is based on the Single Sheet Tester (SST) principle. Two concentric copper coils are wound around the sample. A controlled electrical current is enforced in the primary coil using a Kepco 50/20 bipolar operational amplifier, which will cause a magnetic excitation field in the sheet. The magnetic field will excite the magnetic domains in the ferromagnetic silicon steel. This resulting magnetic flux induces a voltage in the secondary coil which can be measured using a National Instruments data-acquisition system. In order to measure the core losses correctly, the applied excitation current in the primary coil must generate a sinusoidal flux density waveform. Therefore, the MATLAB-based control algorithm iteratively excites the steel and measures the induced voltage in the secondary coil, correcting the excitation current signal until the secondary voltage is sinusoidal. The magnetic flux path is closed using a double ferromagnetic yoke. In Figure 2, a cross section view of this setup is shown. In order to measure the effect of mechanical stress on the magnetic properties of the silicon steel, the SST setup was modified to accomodate an external mechanical load. This was done using a stress bench. The stress bench was constructed using two clamps attached to an aluminium frame, with one clamp fixed to the frame and the other mounted on a threaded axis. Using a rotary handle, the external load on the silicon steel samples between the clamps was controlled. This magnetomechanical measurement setup was custom developed by EELAB at Ghent University. The complete setup is shown in Figure 3.

### 2.3. Tensile Test Setup

**Figure 4:**
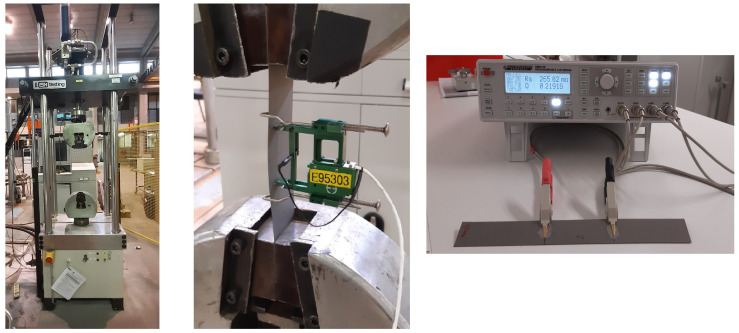
(**Left**): MTS tensile tester setup for measuring the stress strain curve. (**Middle**): extensometer attached to sample in tensile tester. (**Right**): Experimental setup for measuring the electrical resistance using the 4-wire method.

In the reported experiments, the magnetic properties of M270-35A steel are measured at different levels of pre-release strain. This pre-release strain indicates the strain (i.e., the ratio of sample elongation and initial sample length) before the load was released. However, it is common in literature to use the stress-value instead of the strain-value. Therefore, the stress-strain curve is added in the results section of this paper. This way, the reported strain values can be related to the actual externally applied stress values. Stress strain curves for M270-35A were measured in rolling direction using an servo-hydraulic testing machine developed by MTS using MTS793 software. The elongation was measured using a 50 mm extensometer (Figure 4). Traditionally, stress strain curves are obtained using samples having a smaller cross section in the middle because this geometry ensures that the point of fracture is located in the middle of the sample. However, a standard Epstein strip shape was used for tensile testing, which has an equal cross section along the length of the strip. However, the fracture appeared in the middle of the sample, and the obtained curve was in close correspondence with the datapoints reported in the M270-35A data sheet.

### 2.4. Transmission Electron Microscopy Measurements

Using TEM, the microstructure of the material was investigated. For dislocation density measurements, a JEOL JEM2200FS FEG TEM/STEM was used equipped with an in column omega-type energy filter per se electron energy loss spectrometer (EELS), operated under 200 kV. The thickness of the TEM foil was measured in the middle of every image frame in Scanning Transmission Electron Microscopy (STEM) mode using Electron Energy Loss Spectroscopy (EELS). The micrographs obtained from the TEM were used for calculating the dislocation density using a methodology described in [31].

### 2.5. Magnetic Measurement Principles

The measured magnetic flux density is averaged over the single sheet cross section *S*:

(1) $$B_{a}\left(t\right)=\frac{-1}{n_{2}S}\int_{0}^{t}e\left(\tau \right)d\tau$$

with *n*_2_ the amount of secondary turns and *e(t)* the measured induced voltage in the secondary coil. The measured space-average magnetic field strength is derived from Ampères law for closed circuits with homogeneous fields

(2) $$H_{a}=\frac{n_{1}i\left(t\right)}{l_{a}}$$

where *n*_1_ is the amount of turns in the excitation coil, *i(t)* is the measured current in the excitation coil and *l_a_* is the average magnetic path length of 110 mm. The relative magnetic permeability *μ_r_(B)* can be calculated by measuring low frequency (<5 Hz) quasi-static symmetric *BH* loops. The peak values from the *H* - and *B*-waveform for this loop can then be applied for calculating the relative permeability using the constitutive relation *μ_r_(B) = B / (μ_0_H)*. In this paper, the analysis is limited to sinusoidal flux patterns.

The core losses (W/kg) are calculated based on the measured global *B_a_* and *H_a_* using

(3) $$P_{core}=\frac{f}{\rho }\oint H_{a}dB_{a}$$

with *f* the excitation frequency and ρ the mass density. The core losses in each sample were measured in several steps. First, the sample was fixed in the bench. Then, the mechanical load was increased until a specific amount of strain was imposed, and the core losses were measured for a range of frequencies (20, 40, 60, 80, 100, 150, 200, 250, 300, 350 and 400 Hz) and a range of *B_p_* between 0.2 T and 1.2 T with intervals of 0.1 T. Afterwards, the load was released and the core losses were again measured within the same range. The maximum strain imposed was increased between the consecutive samples, ranging from 0% (no plastic deformation) to 10.4%. For each strain level a different sample was used. Each sample which was cut from the same mother coil.

### 2.6. Method of Core Loss Analysis

The method of core loss separation is based on the statistical loss theory, in which the total core losses *P_tot_* can be segregated in hysteresis losses, classical (eddy current) losses and excess losses. These loss components have different physical backgrounds and are manifested at different time and space-scales.

(4) $$P_{tot}=P_{hyst}+P_{cl}+P_{exc}$$

Measuring these losses at different frequencies allows for the extrapolation of the quasi-static hysteresis energy loss per cycle in (J/m^3^)

(5) $$W_{h}\left(B_{p}\right)=\lim_{f\to 0}\frac{P_{tot}\rho }{f}$$

When the applied flux density waveform is sinusoidal, the classical losses can be calculated analytically using in (W/kg)

(6) $$P_{cl}\left(B_{p},f\right)=\frac{B_{p}^{2}f^{2}d^{2}\pi^{2}\sigma }{\rho 6}$$

It can be assumed that the electrical conductivity σ does not change when the material is strained. This was verified in the Results Section 3.2 with electrical resistance measurements. The excess loss can now be derived from (4). A commonly used definition for the excess losses in NGO electrical steel is

(7) $$P_{exc}\left(B_{p},f\right)=8\sqrt{\left(1/\rho \right)GSV_{0}\left(B_{p}\right)}{\left(B_{p}f\right)}^{1.5}$$

where *G* = 0.1357 is a constant. It can be assumed that the parameter *V*_0_ together with hysteresis energy loss *W_h_* could describe the effect of the applied external mechanical stress on the microstructure and on the magnetic properties of NGO electrical steels, where *V*_0_ describes the influence on losses of various microstructural features such as (among others) grain size, crystallographic texture and residual stresses [10].

### 2.7. Electrical Resistance Measurements

When performing the core loss analysis, it was assumed that the classical loss component did not change when the material is plastically deformed. In order to verify this assumption, the electrical resistance of samples with varying levels of plastic deformation was measured. If there is no significant variation in the measured resistance between the different samples, it can be concluded that the resistivity of the material is not significantly impacted by the plastic deformation. The coating on the samples was removed locally in order to improve the electrical contact between the steel and the measurement probes. The probes were connected to the Rohde & Schwarz programmable LCR bridge using the 4-wire method (Figure 4). For each sample, a fixed distance of 7 cm was maintained between the measurement probes. The resistance was measured in the silicon steel between the probes.

## 3. Results

### 3.1. Stress Strain Measurements

**Figure 5:**
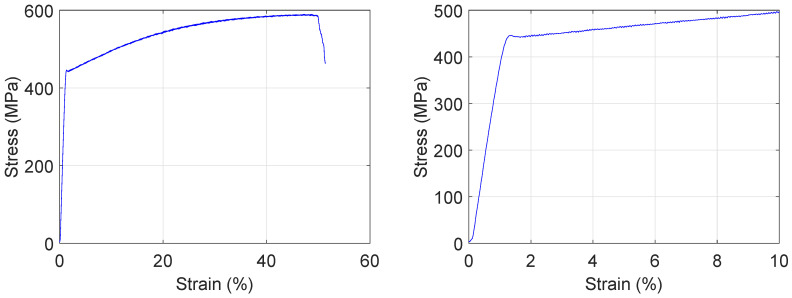
Stress strain curve measured on M270-35A in rolling direction up to the point of fracture (**left**) and below 10% strain (**right**).

Using the MTS tensile tester, the complete stress strain curve was obtained and is displayed on the left hand side in Figure 5. The curve was constructed using approximately 2000 measured datapoints. The M270-35A grade steel has a measured yield strength (0.2% proof) of 452 N/mm^2^ and measured tensile strength of 590 N/mm^2^. The sample fractured at 47.2% elongation. In the following sections, measurements are presented on samples that were strained up to ± 10%. Therefore, the plot on the right hand side of Figure 5 shows the stress strain curve for strain values below 10%. The small decrease and consequent plateau in stress between 1% and 2% strain is caused by the formation of Lüders bands.

### 3.2. Electrical Resistance Measurements

**Figure 6:**
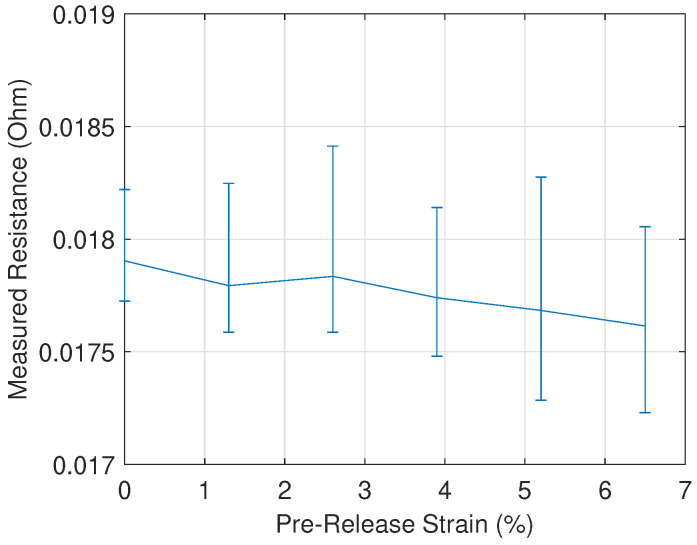
Measured resistance values on strips of M270-35A that underwent increasing levels of plastic deformation. Each measurement was repeated 5 times and the resulting error bars are plotted.

The electrical resistance was measured between two points on the M270-35A samples. The measurement probes were clamped with a fixed distance between them. This was repeated several times for each sample. The electrical resistance was measured on 6 different samples that were strained increasingly up to 5.5% and afterwards released. The variation on each measurement for the same sample allowed the construction of error bars. The results are shown in Figure 6. The average measured resistance of each sample is used to construct the plotted line. When the plastic deformation is increased, the average measured electrical resistance appears to decrease slightly. However, considering the error bars, it can be concluded that there is no significant change in measured resistance when the samples are strained. In the core loss analysis, this proves that the classical loss component can be considered constant for a fixed value of frequency and peak flux density. The classical loss component can be calculated analytically using (6).

### 3.3. Magnetization Curve and Permeability Measurements

**Figure 7:**
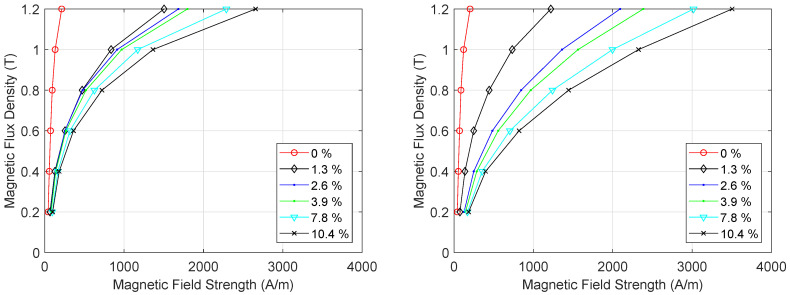
*BH* curves obtained at several levels of pre-release strain from the DD-samples both before (**left**) and after (**right**) the load was released.

The *BH*-curves were measured for 11 samples that were increasingly strained, both before and after the external load was released. A subset of these results is plotted in Figure 7. The strain that elongates the sample negatively impacts the magnetization of the steel. When no external stress is applied, 214 A/m is needed for obtaining a magnetic flux density of 1.2 T in the DD-sample. When the external load is increased and the sample is strained 10.4%, the required magnetic field also increases to 2657 A/m. Afterwards, when the load is released, the sample is plastically deformed without external load, and the required field for obtaining 1.2 T increases further up to 3504 A/m. This value can be found on the right in Figure 7 at 10.4% pre-release strain. This trend also appears for the RD-samples and for the TD-samples.

**Figure 8:**
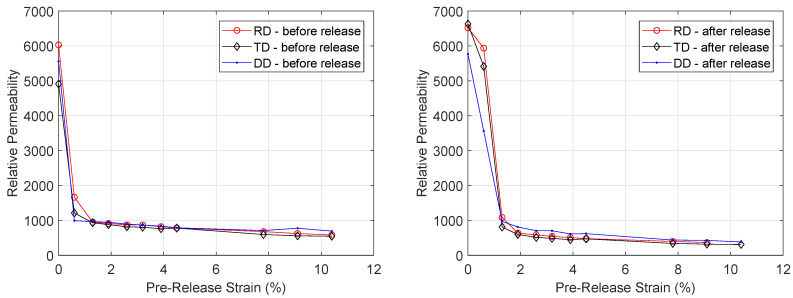
Evolution of relative magnetic permeability at peak magnetic flux density *B_p_* 1 T under increasing levels of strain before (**left**) and after (**right**) release.

The measured *BH* curves can be used for calculating the relative permeability as described in Section 2.5. The relative permeability at 1 T was calculated for three differently cut sets of samples. The results are displayed in Figure 8 and show that the relative magnetic permeability decreases dramatically when the material is strained. When no stress is applied, the relative permeability of the RD sample is approximately 6000. As expected, the DD-sample and TD-sample have a slightly lower relative permeability when no stress is applied. When the samples are plastically deformed, the relative permeability decreases drastically at 10.4% strain to 693, 583 and 546 for the RD, DD and TD-sample respectively. When the external load is released after the sample was strained to 10.4%, the permeability decreased further to 382, 365 and 310 for the RD, DD and TD-sample respectively.

### 3.4. Core Loss Measurements and Loss Separation

**Figure 9:**
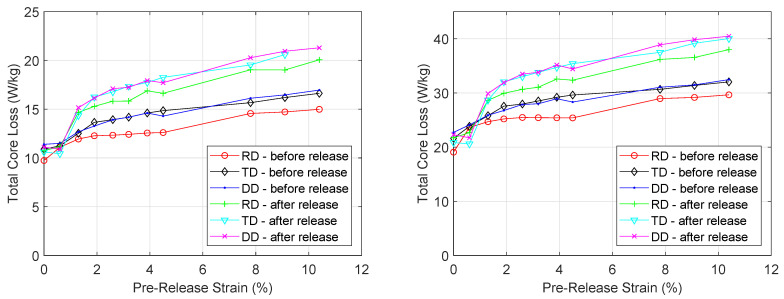
Evolution of total core losses under increasing levels of strain at 200 Hz and *B_p_* = 1.2 T (**left**) and at 400 Hz and *B_p_* = 1 T (**right**).

The effect of plastic deformation both before and after the load release on the magnetic core losses was measured for the RD, TD and DD samples. In Figure 9, the total measured losses are plotted at two excitation levels. When no stress is applied, the total core losses at 400 Hz and 1 T peak flux density are 19.1 W/kg, 22.7 W/kg and 21.6 W/kg for the RD, TD and DD sample respectively. For each sample and excitation level, increasing the plastic strain increased the total core losses. At 10.4% strain, the total core losses increased to 29.7 W/kg, 32.4 W/kg and 32 W/kg for the RD, TD and DD sample respectively. After the load—which enabled the 10.4% strai—was released, the core losses were measured again and it was found that the values further increased up to 37.9 W/kg, 40.4 W/kg and 40 W/kg for the RD, TD and DD sample respectively. Similar to the measured *BH* curves and relative magnetic permeability, the plastic deformation has the same degrading effect on the total core losses for the RD, TD and DD samples.

**Figure 10:**
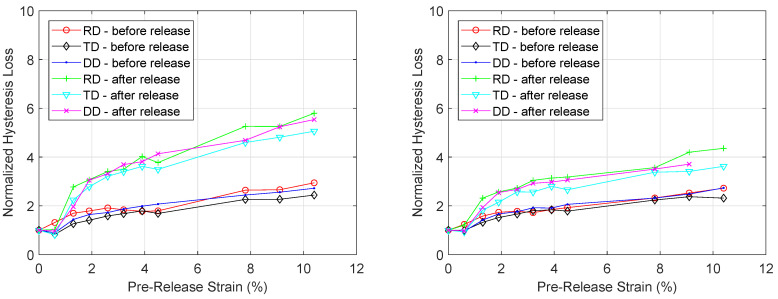
Evolution of hysteresis losses under increasing levels of strain at *B_p_* = 0.4 T (**left**) and at *B_p_* = 1.2 T (**right**). The results are normalized with respect to the hysteresis losses before plastic deformation.

**Figure 11:**
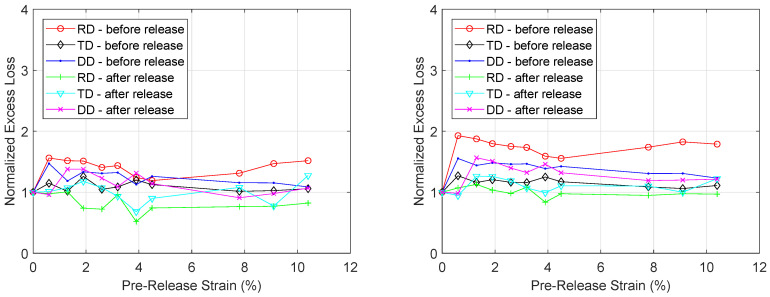
Evolution of excess losses under increasing levels of strain at 100 Hz and *B_p_* = 0.6 T (**left**) and at 400 Hz and *B_p_* = 0.6 T (**right**). The results are normalized with respect to the excess losses before plastic deformation.

Using the technique of core loss separation, the total core losses are broken down into their components and the effect of plastic deformation on each component can be investigated. In Figure 10, the normalized hysteresis loss component at peak flux density 0.4 T and at 1.2 T is plotted for increasing levels of plastic deformation, both before and after the load was released. The same was done for the normalized excess loss component at two excitation levels (100 Hz, 0.6 T and 400 Hz, 0.6 T) in Figure 11. While the effect of increasing plastic strain on the excess loss component is negligible, the effect on the hysteresis loss component is very significant. When no stress is applied, the hysteresis losses at 1.2 T peak flux density are 123 J/m^3^, 163 J/m^3^ and 147 J/m^3^ for the RD, TD and DD sample respectively. For each sample and excitation level, increasing the plastic strain increased the total core losses. At 10.4% strain, the hysteresis losses increased to 335 J/m^3^, 376 J/m^3^ and 401 J/m^3^ for the RD, TD and DD sample respectively. After the load—which enabled the 10.4% strain—was released, it was found that the hysteresis losses values increased further up to 569 J/m^3^, 599 J/m^3^ and 578 J/m^3^ for the RD, TD and DD sample respectively. From these results, it appears the effect of plastic deformation on the hysteresis loss component is the main contributing factor to the observed effect of plastic deformation on the total core losses.

### 3.5. TEM Measurements

**Figure 12:**
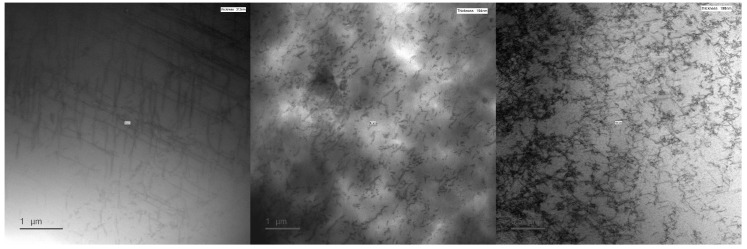
Micrographs obtained using TEM for three samples of M270-35A silicon steel. (**Left**) unstrained sample. (**Middle**) Sample strained to 4.5% and afterwards released. (**Right**) Sample strained to 10.4% and afterwards released.

**Figure 13:**
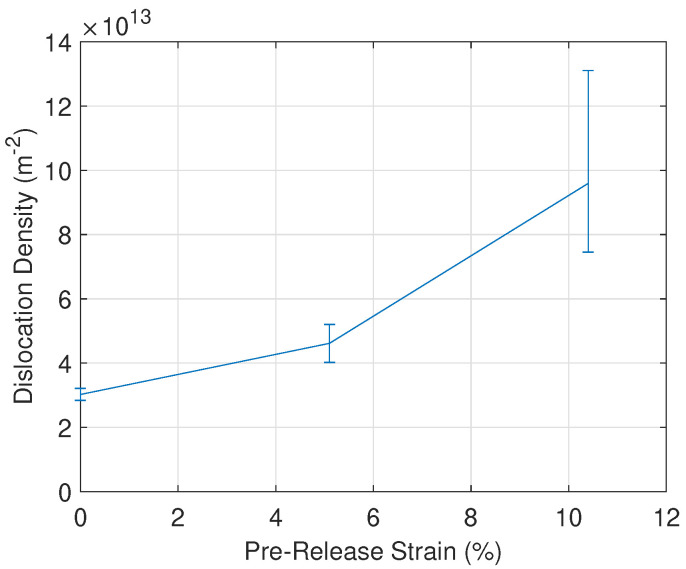
Dislocation density as a function of pre-release strain.

The effect of plastic deformation and load release on the hysteresis loss component is investigated further in this section. Using TEM, the dislocation density in the microstructure of the samples (after load release) can be measured. In Figure 12, micrographs obtained using the TEM are shown for three samples. From these micrographs, the dislocation density was calculated and plotted in Figure 13. The calculation was repeated several times for each sample. The average calculated dislocation density is used for the plot line and the error bars were constructed correspondingly. At 10.4% strain, the variability of the calculations was higher due to the presence of entangled dislocations. From these data, it was confirmed that the dislocation density rises sharply due to plastic deformation. In the unstressed sample, the average dislocation density is 3.2 × 10${}^{13}$/m^2^. The samples that were plastically strained to 5.1% and 10.4% had an average dislocation density of 5.2 × 10${}^{13}$/m^2^ and 1.31 × 10${}^{14}$/m^2^, respectively. The observed increase in dislocation density influences the magnetic properties because the dislocations become entangled, forming stronger pinning sites which impede domain wall motion [32]. This impeded motion causes a degradation of the magnetic properties. Also in [32], the Jiles-Atherton hysteresismodel is used to highlight the relation between several microstructural parameters and the magnetic hysteresis properties. In this model, parameter *k_j_* is proportional to the coercive field and the hysteresis loss component and parameter *a_j_* is inversely proportional to the relative permeability. Both of these parameters are proportional to the square root of dislocation density, which is in correspondence with the measurements reported in this paper.

## 4. Conclusions

The material state near cutting edges and other local plastic deformed areas of ferromagnetic cores is investigated using a magnetomechanical setup. When the material is plastically strained, the *BH* curve is affected and the magnetic permeability is significantly degraded. At 10.4% strain, the permeability has decreased by 88% with respect to the unloaded state for the RD sample. An even further degradation was observed when the plastically deforming mechanical load was released. After load release the permeability has decreased by 94% with respect to the stress- free state. This ‘released’ load state corresponds more closely to the material state near cutting edges of electrical steel sheets. The total core losses were measured and it was found that the plastic deformation significantly increases the core losses, similar to the magnetic degradation observed in the *BH* curves. Core loss analysis has shown that the large increase in core energy losses is mainly caused by a sharp increase of the hysteresis loss component. At 10.4% strain, the hysteresis loss component has increased by 55% with respect to the unloaded state for the RD sample. After mechanical load release, the hysteresis loss component has increased further by 98% with respect to the stress-free state. Both the classical and excess loss component showed no clear changes under increasing deformation. Within the measured range, the electrical resistivity remains unaffected by the sample deformation. The effect of plastic deformation on the magnetic properties is similar for samples cut in different directions. As expected, TEM measurements on unloaded deformed samples have shown a sharp increase in dislocation density due to deformation. At 10.4% strain, the dislocation density has increased approximately 330% with respect to the unloaded state. The effect of plastic strain on the hysteresis loss component and the dislocation density shows a positive correlation. The main results of the paper show that the magnetic properties of electrical steels are significantly degraded when plastically strained and afterwards released. These results indicate the importance of using material models which incorporate the effects of residual stress when developing accurate electrical machine models.

## Abbreviations

The following abbreviations are used in this manuscript:

RD	Rolling Direction
TD	Transverse Direction
DD	Diagonal Direction
NGO	Non Grain Oriented
SST	single Sheet Tester
TEM	Transmission Electron Microscopy
